# Perioperative Risk Assessment and Management in Patients With CKD, Dialysis Dependence, and Kidney Transplantation

**DOI:** 10.1016/j.ekir.2026.107137

**Published:** 2026-10-10

**Authors:** Srinath Yadlapalli, Divya K. Nekkalapudi, Jay B. Wish

**Affiliations:** 1 Division of Nephrology, Indiana University School of Medicine, Indianapolis, Indiana, USA; 2 Division of Rheumatology, Indiana University School of Medicine, Indianapolis, Indiana, USA

**Keywords:** chronic kidney disease, hemodialysis, kidney transplant, perioperative care, peritoneal dialysis

## Abstract

As the prevalence of chronic kidney disease (CKD), end-stage kidney disease, and kidney transplantation continues to rise, these patients are increasingly undergoing elective surgical procedures. This unique population presents significant clinical challenges due to extensive comorbidities, heightened cardiovascular risk, and high susceptibility to acute kidney injury (AKI). In patients with advanced CKD, an episode of perioperative AKI can exhaust renal reserve, often precipitating a transition to acute or chronic dialysis. This review provides a comprehensive framework for perioperative risk assessment from a nephrology perspective. Beyond standard cardiovascular evaluation, we examine kidney-specific nuances, including AKI prediction models for cardiac and non-cardiac surgery and the perioperative management of hypertension and anemia. Furthermore, we detail specialized care for hemodialysis (HD), peritoneal dialysis (PD), and transplant recipients, including the optimal timing of dialysis and the management of perioperative medications. Distinct considerations for PD patients, such as the risk of peritonitis, dialysate leakage, and wound dehiscence following abdominal surgery, are addressed. By integrating these strategies, providers can mitigate complications and improve outcomes in this high-risk surgical cohort.

## Introduction

Over the last 3 decades, chronic kidney disease (CKD) prevalence and mortality have risen significantly.^1^ Epidemiologic evidence underscores the high frequency of surgical intervention in this population.^2^ A large study of over 1.4 million North American adults revealed that individuals with lower estimated glomerular filtration rate (eGFR) undergo major surgery at significantly higher rates than those with preserved kidney function. This surgical burden is particularly pronounced in dialysis-dependent populations; for instance, the incidence rate of major surgery for men under age 65 receiving dialysis is 243.8 per 1000 person-years, a 13-fold increase over the G1-G2 cohort.^3^

The perioperative mortality hierarchy among individuals with kidney disease mirrors their known underlying mortality risks, with the greatest risk observed in those on hemodialysis (HD), followed by individuals on peritoneal dialysis (PD), those with advanced CKD (stages 4–5), and transplant recipients.^4^, ^5^, ^6^, ^7^ This heightened risk is driven by systemic comorbidities and an increased incidence of major adverse cardiovascular events, which classically comprise myocardial infarction, stroke, and cardiovascular death.^8^ Furthermore, postoperative morbidity, including unplanned reoperation, stroke, bleeding, pulmonary complications, infections, and thromboembolic events, is significantly higher in advanced CKD.^4^

Postoperative acute kidney injury (AKI) is a critical threat, often precipitated by intraoperative hypotension, occurring in 9% of noncardiac surgeries.^9^ AKI increases mortality, intensive care unit stays, and healthcare costs.^10^, ^11^ Beyond the immediate postoperative period, AKI predicts poor long-term outcomes, such as the development of *de novo* CKD, accelerated progression of existing CKD toward kidney failure, and increased risk of cardiovascular events and rehospitalization. Collectively, these factors exacerbate mortality, morbidity, and the economic burden on healthcare systems.^12^

Although preoperative cardiac and pulmonary optimization are entrenched in surgical culture, kidney-specific strategies remain underused despite their profound impact.^10^ This clinical gap highlights the need for the development of perioperative nephrology as a formal, multidisciplinary field.

## Scope of Review

This review focuses on perioperative risk assessment for postoperative AKI in CKD, alongside the management of CKD, HD, PD, and kidney transplant recipients. Drawing on the authors’ background as internists running perioperative clinics and transitioning to nephrology, the scope is tailored to the practical needs of general internists, nephrologists, and anesthesiologists. Although related topics such as cardiac risk assessment, anesthesia selection, and frailty are touched upon, only their most critical aspects are explored. Broader subjects like detailed cardiac investigations, major cardiovascular events, infections, bleeding, and functional decline fall outside the intended scope.

## Methodology for Literature Review

As a clinical narrative review, a formal systematic review protocol was not used. Instead, targeted searches were conducted in PubMed, MEDLINE, and Google Scholar using structured keyword combinations covering AKI, perioperative risk prediction models, surgery types, CKD, and specialized populations (HD, PD, and kidney transplantation). Literature selection was guided by clinical relevance and methodological strength, prioritizing randomized controlled trials published within the last decade, alongside professional society guidelines and position statements from Kidney Disease: Improving Global Outcomes (KDIGO) and the American Heart Association to ensure standard-of-care alignment.

## Definition

Postoperative AKI is diagnosed using KDIGO criteria within 7 days of surgical intervention, defined by an absolute serum creatinine increase of at least 0.3 mg/dl within 48 hours, a 50% increase within 7 days, or oliguria for 6 hours or more.^13^

## Perioperative Risk Assessment Factors

Perioperative risk assessment in patients with kidney disease should include procedure-specific risk and patient-specific risk. Procedure-specific risk encompasses both cardiovascular and AKI risk assessment based on the type of surgery and duration of anesthesia. Synthesizing these stratifications establishes the overall risk profile, directly guiding clinical decision-making, such as goals of care discussions and decisions on whether to proceed with surgery. Although these scores help frame overall risk, detailed evaluations regarding further cardiac investigations are beyond the scope of this review.

### Procedure-Specific Risk

#### Type of Surgery

First, providers must determine the risk level of the surgical procedure based on both cardiovascular and kidney-specific criteria. Regarding cardiovascular risk, the 2022 European Society of Cardiology guidelines categorize procedures by their 30-day risk of major adverse cardiovascular events into low (< 1%), intermediate (1–5%), and high risk (> 5%).^14^ Alternatively, the 2024 American Heart Association guidelines simplify this into low risk (< 1%) and elevated risk (≥ 1%).^15^

Beyond cardiovascular stratification, procedures must be evaluated for their propensity for AKI. Although no universal consensus exists for AKI risk stratification, we propose that procedures can be broadly delineated into low and elevated risk categories based on existing evidence (Table 1). These categories serve only as a baseline; the true risk of AKI is more heavily influenced by perioperative factors, such as the severity and duration of hypotension, preoperative anemia, blood loss, transfusion requirements, vasopressor use, and surgical re-exploration, than by the procedure type alone.^16^, ^17^, ^18^, ^19^, ^20^, ^21^, ^22^

**Table 1:** Surgical risk stratification for cardiovascular outcomes (per 2022 ESC Guidelines) and kidney outcomes

	Risk categories for cardiac and kidney based on surgical procedures	
Cardiac risk	Low surgical risk (< 1%)	Elevated Risk
	Ophthalmic surgery Dental Breast Minor urological surgery Minor gynecological procedures Minor orthopedic	Intermediate surgical risk (1–5%)
		Asymptomatic carotid endarterectomy Head and neck surgery Major orthopedic surgery (e.g., hip and spine surgery), Major urological Major gynecological procedures kidney transplantation
		High surgical risk (> 5%)
		Symptomatic carotid surgery Aortic and major vascular surgery Bowel perforation repair and duodeno-pancreatic surgery Liver resection Lung transplant Liver transplant
Kidney risk	Low risk for AKI	Elevated risk for AKI
	Ophthalmic surgery Dental Minor urological surgery (e.g., TURP) Minor gynecological procedures Minor orthopedic (e.g., meniscectomy)	Major cardiac surgeries such as CABG and valvular repairs Major vascular surgeries Major thoracic surgeries Intraperitoneal surgeries (especially open laparotomy) Major neurosurgical procedures Emergency procedures

AKI, acute kidney injury; CABG, coronary artery bypass grafting; ESC, European Society of Cardiology; TURP, transurethral resection of the prostate.

#### Anesthesia Type and Duration

Second, providers must consider anesthesia type and duration, as both general and neuraxial (spinal or epidural) modalities can cause hypotension.^23^ Although some studies suggested neuraxial anesthesia carries lower AKI risk,^24^, ^25^ larger randomized trials such as the Regional versus General Anesthesia for Promoting Independence after Hip Fracture found no statistically significant difference, with AKI occurring in 4.5% of the spinal group versus 7.6% for the general anesthesia group.^26^ Ultimately, intraoperative hemodynamic instability, specifically the severity and duration of hypotension, the requirement for perioperative vasopressors, and the balancing of fluid administration against blood and insensible losses, are more critical determinants of kidney outcomes than the anesthetic modality alone.^9^, ^27^, ^28^ Similarly, the 2024 American Heart Association guidelines deem either approach reasonable, as cardiovascular event rates are comparable.^15^

### Patient-Specific Risk

Third, evaluate individual cardiac risk using the Revised Cardiac Risk Index (RCRI) or the American College of Surgeons National Surgical Quality Improvement Program calculator. A preoperative serum creatinine > 2 mg/dl increases a patient's RCRI score. The American College of Surgeons National Surgical Quality Improvement Program incorporates more variables, including procedural type and functional status, while explicitly including dialysis and stage 2 or 3 AKI as risk factors. A significant advantage of the American College of Surgeons National Surgical Quality Improvement Program is its ability to estimate other complications, including postoperative kidney failure.^29^

Though these risk predictors are frequently used in patients with kidney disease, it is important to understand their limitations in this population. The RCRI uses preoperative serum creatinine rather than eGFR, which may be an unreliable marker in patients with advanced kidney disease. Furthermore, its binary serum creatinine threshold (> 2 mg/dl) provides limited discrimination across a heterogeneous population with advanced CKD and kidney failure. Notably, validation studies have shown that the RCRI performs poorly in this cohort, significantly overestimating the risk of major adverse cardiovascular events in patients requiring chronic kidney replacement therapy.^30^, ^31^, ^32^ Similarly, though the American College of Surgeons National Surgical Quality Improvement Program calculator includes a variable for dialysis, it does not differentiate between patients based on chronic versus temporary dialysis, dialysis modality, dialysis vintage, recent dialysis-related instability, or residual kidney function.

### Frailty and Shared Decision Making

Frailty is independently associated with worse postoperative complications, increased length of stay, and higher mortality.^33^, ^34^ The prevalence of frailty, sarcopenia, and poor functional status is high in both CKD and kidney failure. Sarcopenia and poor functional status are associated with increased falls, hospitalizations, and adverse perioperative outcomes.^35^, ^36^, ^37^, ^38^, ^39^, ^40^ Furthermore, cognitive impairment, when coupled with these markers of physical vulnerability, significantly worsens long-term outcomes.^41^, ^42^ These vulnerabilities should be taken into consideration while deciding whether to proceed with surgery. When surgery is considered, clinicians and patients must use a robust shared decision-making framework to discuss anticipated perioperative complications, length of stay, and mortality. Crucially, discussions should elicit patient priorities regarding quality of life, intensive care admission, prolonged hospitalization, desired hospital-free survival, temporary or permanent dialysis, functional decline, and postoperative rehabilitation requirements. Ultimately, conversations must center on aligning patient goals, including patient preferences for kidney replacement therapy versus comprehensive conservative therapy before proceeding.^43^, ^44^, ^45^

## AKI Prediction Models

### Methodology for AKI Risk Prediction Models

Numerous AKI risk prediction models exist in literature. For this narrative review, we selected specific models based on the following 3 criteria: a high total sample size combining the derivation and validation cohorts (*N* ≥ 30,000), external validation, and the availability of a risk index and/or score or online risk calculator designed for practical use by clinicians at the bedside. Based on these criteria, we narrowed our focus to the following 4 models: the Thakar *et al.*^46^ and Demirjian *et al.*^47^ indices for cardiac surgery, the Kheterpal *et al.*^48^ and simple postoperative AKI risk (SPARK; Park et al., 2019)^49^ indices for noncardiac surgery. We acknowledge that many other models are available, but this review focuses on these 4 widely validated tools as primary references for clinical risk stratification. Key components, clinical strengths, and limitations for each of these models are summarized in Table 2.

**Table 2:** Comparison of validated risk prediction models for perioperative AKI

Model and surgery category	Key risk factors (points)	Primary outcome/ROC and risk categories	Clinical application & utility
Thakar model (cardiac surgery)	Female gender (1), CHF (1), LVEF < 35% (1), Preoperative use of IABP (2), COPD (1), Insulin-requiring diabetes (1), Previous cardiac surgery (1), Emergency surgery (2); Surgery type: Valve only (1), CABG + Valve (2), Other cardiac surgery (2); Pre-op Creatinine (mg/dl): 1.2 to < 2.1 (2), ≥ 2.1 (5)	Primary outcome: AKI-D Risk Score (Frequency of AKI-D): • 0–2 (0.4%) • 3–5 (1.8%) • 6–8 (7.8%) • 9–13 (21.5%) ROC: 0.81	Strengths: - Excellent for predicting severe AKI (AKI-D) - Best for preoperative counseling and shared decision-making. Limitations: - Poor discrimination against early-stage AKI - It lacks dynamic capability - It tends to underestimate AKI-D in some cohorts
Cleveland clinic (cardiac surgery)	Postoperative Labs: Serum sodium, potassium, bicarbonate, BUN and albumin. Dynamic Markers: Perioperative creatinine change. Temporal Data: Duration from end of surgery to metabolic panel draw	Primary outcome: Moderate-to-severe AKI (KDIGO Stage ≥ 2) and AKI requiring dialysis within 72 h or 14 d after surgical procedure Risk Score: Calculated as a dynamic risk probability based on real-time postoperative laboratory trends. ROC: AKI stage ≥ 2: • ROC: 0.88 (72 h) • ROC: 0.85 (14 d) AKI-D: • ROC: 0.92 (72 h) • ROC: 0.90 (14 d)	Strengths: - Dynamic, facilitates real-time postoperative risk updates, enabling timely interventions (e.g., volume optimization, avoidance of nephrotoxins/hypotension) - Predicts moderate to severe AKI in addition to AKI-D Limitations: - It tends to overestimate the risk of AKI-D in high-risk categories.
General surgery index (non-cardiac)	Age ≥ 56 (1), Male sex (1), Acute CHF (1), Ascites (1), Hypertension (1), Emergency surgery (1), Intraperitoneal surgery (1), Diabetes mellitus -oral or insulin therapy (1), Pre-op Creatinine > 1.2 mg/dl (1)	Primary outcome: AKI within 30 d (Creatinine increase ≥ 2.0 mg/dl or new-onset dialysis) Risk class (number of risk factors) and AKI incidence Class I (0–2): 0.2% Class II (3): 0.8% Class III (4): 2% Class IV (5): 3.6% Class V (6+): 9.5% C-statistic: 0.80	Strengths: - Offers a simple, point-based system for rapid bedside application without the need for complex calculators. Limitations: - AUC ranges (0.65–0.79) in external validation - Tendency to underestimate AKI risk in diverse populations
SPARK index (non-cardiac)	Patient factors: Low eGFR (up to 22), Advanced age (up to 13), Male (8), Hypoalbuminemia (8), Anemia (4), Diabetes (4), Hyponatremia (3) Surgical Factors: Expected duration (5 per hour), Emergency surgery (7), RAAS blocker use (6), Albuminuria (6)	Three-tier ordinal outcome: 1. No AKI 2. Low stage (Stage 1) 3. Critical AKI (Stage ≥ 2, death, or dialysis within 90 d) Risk Score: Class A • Score < 20 • AKI < 2% • Critical AKI < 2% Class B • Score 20–39 • AKI ≥ 2% • Critical AKI < 2% Class C • Score 40–59 • AKI ≥ 10% • Critical AKI ≥ 2% Class D • Score ≥ 60 • AKI ≥ 20% • Critical AKI ≥ 10% C-statistic: 0.72	Strengths: - Heavily weights markers of CKD (eGFR, albuminuria, hypoalbuminemia, RAAS inhibitor use) - Four-tier framework supports targeted preventive care and risk-based monitoring Limitations: - Tends to overestimate risk in high-risk categories - Suboptimal discriminative capacity in neurosurgery and urology cohorts

AKI, acute kidney injury; AKI-D, acute kidney injury requiring dialysis; BUN, blood urea nitrogen; CABG, coronary artery bypass grafting; CHF, congestive heart failure; CKD, chronic kidney disease; COPD, chronic obstructive pulmonary disease; eGFR, estimated glomerular filtration rate; IABP, intra-aortic balloon pump; KDIGO, Kidney Disease: Improving Global Outcomes; LVEF, left ventricular ejection fraction; RAAS, renin-angiotensin-aldosterone system; ROC, receiver operating characteristic.

### AKI After Cardiac Surgery

#### Thakar Model

The Thakar model was derived from a large-scale retrospective cohort of 31,000 patients at the Cleveland Clinic to predict the incidence of AKI requiring dialysis (AKI-D).^46^ It should be noted that preoperative creatinine ≥ 2.1 mg/dl, insulin requiring diabetes, and congestive heart failure overlap with the RCRI score. A patient with these comorbidities undergoing coronary artery bypass grafting would be high-risk for both major adverse cardiovascular events and AKI. It showed a robust area under the receiver operating characteristic curve of 0.81, with internal validation yielding a receiver operating characteristic of 0.82. Subsequent validation studies have consistently confirmed the model’s strong discriminative capacity. In a study comparing eight prediction models for cardiac surgery-related AKI, the Thakar model was found to have the best predictive value.^50^, ^51^, ^52^, ^53^, ^54^

#### Cleveland Clinic Risk Calculator

The Cleveland Clinic risk calculator incorporates postoperative variables, perioperative creatinine changes, and the precise duration from surgery to blood draw to provide risk estimates for both stage 2 and/or 3 AKI and AKI-D. Because absolute creatinine values often lag behind the initial insult, this multifaceted approach allows for earlier detection of AKI. The clinical accuracy of this dynamic strategy is high, with an area under the receiver operating characteristic curve of 0.91 for predicting dialysis within 72 hours, and 0.90 within 14 days post-surgery.^47^ External validation has confirmed these robust findings, with reported area under the curve (AUC) values for AKI-D ranging from 0.87 to 0.92.^55^

### AKI After Noncardiac Surgery

#### General Surgery AKI Risk Index

Several models predict AKI risk in noncardiac surgery.^56^ The general surgery AKI risk index uses various clinical and procedural variables to categorize patients into five classes based on the number of risk factors: Class I (0–2), II (3), III (4), IV (5), and V (≥ 6). Class V patients face a 9% AKI risk. Although the index achieved a robust c-statistic of 0.80 with consistent internal validation,^48^ external validation studies report variable discriminative capacity, with AUC values ranging from 0.65 to 0.79.^57^, ^58^, ^59^

#### SPARK index

The SPARK index integrates preoperative eGFR with several key perioperative factors to stratify patients into 4 risk classes, ranging from Class A (low risk) to Class D (high risk for critical AKI). Critical AKI includes KDIGO AKI stage ≥ 2, post-AKI death, or dialysis within 90 days.^49^ Although the SPARK index achieved a c-statistic of 0.80 in its discovery cohort, performance decreased to 0.72 upon external validation. Furthermore, a Japanese cohort study reported that while AKI incidence increased in alignment with SPARK risk classes, the index overestimated the absolute probability of AKI in high-risk categories, leading to suboptimal calibration and discrimination.^60^ In another study evaluating patients admitted to the intensive care unit after noncardiac surgery, the SPARK index demonstrated fair discrimination and calibration.^61^ Notably, a comparative assessment of 9 noncardiac surgery models identified the SPARK index as providing a positive net clinical benefit, despite modest overall discrimination.^62^

### CKD-Specific Limitations

Although patients with kidney disease were included in original derivation cohorts, none of these prediction models were developed specifically for CKD. A primary limitation is their reliance on serum creatinine, which is heavily influenced by muscle mass and sarcopenia, and inherently lags behind acute physiological insults. General models also omit CKD-specific variables like chronic inflammation, hypoalbuminemia, mineral bone disorder, and baseline anemia, which compound vulnerability to hemodynamic fluctuations. Consequently, model calibration in CKD requires validation—though general tools like the Thakar score show a c-index of 0.78, models specifically developed for CKD achieve higher discrimination (e.g., Wang *et al.*, c-index 0.81).^63^ Although emerging machine learning models incorporate eGFR, albumin, hemoglobin, and electrolytes to address these gaps, they require rigorous prospective validation.^64^

### Clinical Application

Although these models incorporate varying parameters and lack universal implementation criteria, their utility is rooted in patient physiological vulnerability. Reduced kidney reserve renders patients with CKD highly susceptible to hemodynamic fluctuations. Conditions like diabetes and heart failure drive renin-angiotensin-aldosterone system (RAAS) activation,^65^ while heart failure-induced venous congestion further compromises trans-kidney pressure gradients.^66^

Clinicians must interpret these tools with caveats—scores complement clinical assessment, calibration is poor in advanced CKD, validation across advanced CKD and transplantation is lacking, and evidence that score-guided interventions improve hard outcomes remains limited. Recognizing these limitations, in the setting of cardiac surgery, stage 3 CKD or greater, especially when compounded by congestive heart failure or insulin-requiring diabetes, can prompt consideration of the Thakar score. For patients who have already undergone cardiac surgery, the Cleveland Clinic risk calculator can assist in guiding informed, real-time prognostic discussions with patients. Similarly, in noncardiac surgery, the SPARK or General Surgery index may be used for advanced CKD in older patients with diabetes and congestive heart failure undergoing intraperitoneal or prolonged procedures.

Ultimately, the value of these models lies in supporting targeted perioperative interventions. Identifying high-risk patients should prompt kidney-protective strategies and a low threshold for early nephrology consultation. Although emergency surgery inherently limits the window for optimization, elective and urgent procedures provide a critical opportunity to balance cardiac and kidney risks through pre-procedural planning.

## Perioperative Management of Anemia

Both CKD and kidney failure are associated with increased perioperative bleeding requiring blood transfusions.^67^, ^68^ Presurgical anemia treatment is essential, particularly for kidney transplant candidates, as transfusions may lead to human leukocyte antigen sensitization and limit future graft options.^69^ Furthermore, preoperative anemia is a recognized risk factor for AKI and mortality.^70^

According to the 2026 KDIGO anemia guidelines, i.v. iron is preferred for patients on HD with ferritin ≤ 500 ng/ml and transferrin saturation ≤ 30%, whereas patients not undergoing dialysis and those on PD require repletion if ferritin < 100 ng/ml and transferrin saturation < 40%, or if ferritin is 100 to 300 ng/ml with a transferrin saturation < 25%, using oral or i.v. routes tailored to patient preference, severity, tolerability, and cost, recognizing that these recommendations rely on very low certainty evidence.^71^ Iron stores should be repleted before initiating erythropoiesis-stimulating agents or hypoxia-inducible factor-prolyl hydroxylase inhibitors. The target hemoglobin for patients with CKD and kidney failure on dialysis is 10 to 11 g/dl. Erythropoiesis-stimulating agents are generally not recommended to increase hemoglobin to > 11 g/dl because of increased risks of stroke and cardiovascular events.^72^

Red blood cell transfusion requires an individualized approach. No universal hemoglobin threshold exists; decisions should be guided by symptoms, active blood loss, urgent surgical requirements, or acute myocardial ischemia. Clinical evidence indicates that a restrictive transfusion strategy (targeting 7 to 8 g/dl) is as safe as a liberal strategy (targeting 9 to 10 g/dl), supporting a more judicious approach.^71^, ^73^ For these patients, clinicians should implement a patient blood management strategy built on the following 3 pillars: managing preoperative anemia, minimizing perioperative bleeding, and addressing postoperative anemia. Tactics include treating underlying causes, optimizing cardiopulmonary status, conducting medication reviews, and minimizing blood loss from diagnostic draws.^74^, ^75^ These efforts are often facilitated by multidisciplinary preoperative anemia clinics, which provide targeted administration of i.v. iron and erythropoiesis-stimulating agents to improve perioperative outcomes.^76^

## Perioperative Management of Patients with CKD not on Dialysis

### Preoperative Planning

Assess kidney risk using AKI prediction models based on the type of procedure. For those with advanced CKD or high AKI risk scores, perform preoperative counseling to align procedural expectations with patient goals. Baseline evaluation requires a kidney function panel and complete blood count to proactively identify and manage electrolyte and/or acid-base derangements and anemia. Ensure blood pressure control, postponing elective procedures for patients with uncontrolled hypertension. Discontinue sodium-glucose cotransporter-2 (SGLT2) inhibitors 3 to 4 days, and diuretics, metformin, or aminoglycosides 48 to 72 hours before surgery.^77^ RAAS inhibitors (RAASi), SGLT2 inhibitors, and glucagon-like peptide-1 (GLP-1) receptor agonists warrant specific perioperative management considerations as detailed below.

#### Renin Angiotensin Inhibitors and Blood Pressure

Although 2024 KDIGO guidelines recommend withholding RAASi for 48 to 72 hours before surgery based on limited historical data,^77^ 2 recent large randomized controlled trials have clarified this dilemma for patients with CKD. The Stop-or-Not trial evaluated RAASi continuation versus discontinuation, while the POISE-3 kidney substudy examined a hypotension-avoidance strategy (including holding RAASi) versus a hypertension-avoidance strategy (Supplementary Table S1). Neither trial reported an AKI benefit from withholding these agents.^78^, ^79^

Synthesizing these contemporary trials, mitigating intraoperative hypotension, and preventing postoperative AKI are distinct clinical objectives. Holding RAASi effectively reduces intraoperative hemodynamic instability and vasopressor requirements, but this hemodynamic benefit does not automatically translate to lower AKI incidence. Consequently, it is our clinical perspective that perioperative RAASi management must be individualized. Clinicians should weigh the proven reduction in intraoperative hypotension against the potential risks of postoperative hypertension and heart failure exacerbation, rather than applying a blanket policy aimed solely at preventing AKI.

In patients with kidney disease, distinguishing between absolute and relative hypotension is critical, particularly with advanced vascular disease, diabetes, or impaired autoregulation.^80^ Standard absolute mean arterial pressure thresholds may overlook kidney ischemia in patients with right-shifted autoregulatory curves. Although the specific threshold for relative hypotension remains debated, clinical consensus emphasizes that reductions from baseline are as physiologically relevant as absolute drops. Therefore, hemodynamic management should consider both absolute values and the magnitude of change from the patient’s habitual blood pressure.^81^, ^82^

#### SGLT2 Inhibitors

Perioperative management requires a pragmatic, risk-stratified strategy. For elective procedures, these agents should ideally be withheld 3 to 4 days before major surgeries and held on the day of surgery for lower-risk procedures. Although the risk of euglycemic diabetic ketoacidosis is heightened in emergency surgery, data regarding postoperative incidence remain varied.^83^, ^84^, ^85^ In emergency settings where withholding is impossible, heightened clinical vigilance remains necessary. Key risk factors for euglycemic diabetic ketoacidosis include a previous history of diabetic ketoacidosis, severe endogenous insulin deficiency, concurrent GLP-1 receptor agonist use, and prolonged perioperative fasting, particularly during high-risk interventions such as cardiac, bariatric, or major colorectal surgeries. In such scenarios, perioperative blood ketone monitoring serves as an essential safety measure.^86^

Clinicians must also account for volume depletion and hypotension, because the natriuretic and diuretic properties of SGLT2 inhibitors can compound intraoperative hemodynamic instability and the risk of AKI under anesthesia. Furthermore, these agents hold limited applicability in patients receiving maintenance dialysis, given the lack of cardiorenal or glycemic indications in kidney failure patients. Finally, postoperative recommencement should be strictly deferred until the patient is hemodynamically stable, has resumed normal oral intake, and any acute perioperative kidney risk has resolved.

#### GLP-1 Receptor Agonists

Perioperative management of GLP-1 receptor agonists balances delayed gastric emptying and aspiration risk against metabolic consequences. Recent consensus guidance has moved away from mandatory cessation toward a risk-stratified, shared decision-making model. In most clinical scenarios, patients may safely continue therapy without a default requirement to hold the agent.

Clinicians should identify high-risk patients by evaluating dose-escalation phases, higher dosing regimens, symptoms, and gastroparesis. For these individuals, mitigation strategies such as a 24-hour preoperative clear liquid diet, point-of-care gastric ultrasound to assess stomach contents, or modified anesthetic induction techniques are preferred over routine discontinuation to preserve long-term cardio-kidney protection.^87^, ^88^, ^89^, ^90^

### Intraoperative and Immediate Perioperative Considerations

Verify medication hold adherence and assess volume status. Confirm baseline hemodynamic stability before the procedure. Employ balanced fluid strategies to manage insensible and operative losses while avoiding fluid overload. Prioritize the maintenance of hemodynamic stability to avoid perioperative hypotension, tailoring targets to the patient’s baseline blood pressure.

### Postoperative Management

Monitor electrolytes and avoid nephrotoxic agents. Use dynamic prediction tools, such as the Cleveland Clinic score, to assess AKI risk. Resume RAASi and SGLT2 inhibitors only after the patient has achieved hemodynamic stability, resumed adequate oral intake, and resolved acute surgical issues.

## Perioperative Management of Patients on HD

### Preoperative Planning

Optimize electrolytes, specifically potassium and bicarbonate, before elective surgery. Establish and achieve a dry weight via targeted ultrafiltration. Control blood pressure, accounting for how volume overload contributes to hypertension in this population. Coordinate dialysis scheduling to align with the surgical window, prioritizing dialysis within 24 hours of surgery, which may include carefully selected same-day sessions, after considering ultrafiltration needs, hemodynamic stability, and electrolyte status.

### Intraoperative and Immediate Perioperative Considerations

Timing of HD relative to surgery significantly impacts outcomes. A large cohort study reported that increasing the interval between HD and surgery is associated with higher 90-day postoperative mortality. Compared with a 1-day interval, a 2-day delay yielded an adjusted hazard ratio of 1.14, whereas a 3-day delay increased the adjusted hazard ratio to 1.25. Patients who received same-day HD had lower mortality risk (adjusted hazard ratio: 0.88; 95% confidence interval: 0.84–0.91) compared with those who did not.^91^

Given these findings, performing HD within 24 hours of surgery helps optimize metabolic and volume status. If same-day dialysis is not logistically feasible, it should be scheduled for the previous day. To account for the risks associated with long interdialytic intervals, clinicians should factor dialysis schedules into elective surgery planning, such as considering non-Monday options for patients on a Monday-Wednesday-Friday regimen when logistically possible. To minimize bleeding risks during the procedure, reduce the dose of heparin administered during the preceding dialysis session or perform dialysis without anticoagulation. Specific clinical trials evaluating the optimal time interval between heparinization during dialysis and surgery are lacking.

Even with a functioning fistula or graft, some centers require a temporary HD catheter if continuous kidney replacement therapy is indicated. Subclavian central venous catheters must be avoided because of the high risk of central vein stenosis, which can compromise the future viability of permanent arteriovenous access. Strictly protect vascular access by prohibiting blood pressure measurements and venous draws on the affected extremity. Finally, avoid morphine in advanced CKD and kidney failure. The accumulation of its kidney-excreted metabolite, morphine-6-glucuronide, risks prolonged respiratory depression, so fentanyl or hydromorphone are preferred alternatives.^92^

### Postoperatively

Monitor electrolytes and volume status closely. Maintain vigilant hemodynamic monitoring to prevent hypotension by accurately tracking intake and output, including surgical blood loss, drain output, and accounting for insensible losses. Adjust ultrafiltration rates accordingly to avoid excessive intravascular volume depletion. Monitor daily weights to assess variations from the established target weight. For dialysis patients with residual kidney function, it is crucial to preserve this by avoiding nephrotoxins, even though they are already classified as having kidney failure. In patients on HD, residual kidney function is associated with significant benefits, including improved volume status and fluid balance, better nutritional status, and enhanced clearance of solutes.^93^

## Perioperative Management of Patients on PD

### Preoperative Planning

Management depends on the surgical site and procedure; preoperative transfer to HD is not routinely necessary, as studies have shown no significant difference in mortality or long-term survival between patients who continue PD and those transitioned to HD.^94^ Address blood pressure, volume status, and electrolyte derangements, particularly hypokalemia. Depending on the surgery, determine the necessity of a temporary transition to HD.

### Intraoperative and Immediate Perioperative Considerations

Although continuing PD is safe, vigilant care is required to mitigate peritonitis risk. The International Society for PD recommends antibiotic prophylaxis for PD catheter placement, colonoscopies, and invasive gynecological procedures. Additionally, any PD patient receiving antibiotics for any indication should also receive antifungal prophylaxis to prevent fungal peritonitis. The abdomen should be kept dry (completely drained of peritoneal dialysate) before endoscopic gastrointestinal and gynecological procedures. To minimize peritonitis risk, clinicians should address modifiable factors, such as hypokalemia and constipation, and consider limiting the use of histamine-2 receptor antagonists.^95^ Small intestinal bacterial overgrowth caused by gastric acid suppression, microbial dysbiosis, and impaired immune function are potential causes of histamine-2 receptor antagonist-associated peritonitis. Interestingly, this risk is not observed with proton pump inhibitors, likely because of the reduced metabolism of histamine-2 receptor antagonists in patients on PD compared with proton pump inhibitors.^96^ Although observational data suggest an association between histamine-2 receptor antagonists and enteric peritonitis, the evidence remains of low certainty, and routine substitution with proton pump inhibitors is unsupported given separate risks like *Clostridioides difficile* infection.

### Postoperative Management

Management of patients on PD is dictated by the surgical site and the nature of the procedure. In cardiothoracic surgery, PD can typically be continued, provided there is no accidental diaphragmatic transgression by chest tubes.^97^ In contrast, management for abdominal surgery is more complex. The decision to transition to temporary HD versus continuing PD, as well as the timing of PD resumption, depends on multiple factors including procedure type, location, surgical approach (open vs. laparoscopic), the potential infectious load, and the patient’s residual kidney function. These decisions should be individualized and require a multidisciplinary approach involving the patient, surgeon, and nephrologist. Concerns regarding inadequate drainage, wound dehiscence, infections, scrotal edema, and dialysate leaks often prompt a conversion to HD.^98^ Many of these complications stem from increased intra-abdominal pressure , which is highly position dependent. Intra-abdominal pressure is lowest when supine and highest when sitting; notably, intra-abdominal pressure in an upright position without dialysate nearly equals the pressure of 2 liters of intraperitoneal fluid in a supine patient. Postoperatively, measures to decrease intra-abdominal pressure, such as using frequent, low-volume fills or performing PD exclusively in the supine position, should be implemented for the first 1 to 2 weeks.^99^, ^100^ Additionally, a strict bowel regimen is essential to prevent constipation-related catheter dysfunction and peritonitis, especially if using opioids.

In patients on PD, measures to preserve residual kidney function are vital, as it provides a clear survival benefit. A reanalysis of the CANUSA study showed that each additional 250 ml of daily urine output was associated with a 36% decrease in relative mortality risk.^101^

## Perioperative Management of Kidney Transplant Patients

### Preoperative Planning

With the increasing number of kidney transplant recipients and their improved survival, more transplant patients are presenting for nontransplant surgeries, even in nontransplant centers. Kidney transplant recipients have increased mortality and odds of AKI compared with nontransplanted counterparts undergoing elective surgery.^102^ Assessment must focus on baseline kidney function, current immunosuppression, and the presence of rejection or active infection.^103^ History should include the date of transplant, and the original cause of kidney failure, especially if that failure resulted from a systemic disease. Previous rejection episodes, the current immunosuppressive regimen, and any history of opportunistic infections should be ascertained. Because immunosuppression intensity and rejection risk are highest in the months immediately following transplant during the transition to stable maintenance levels, elective surgeries should ideally be delayed until graft function and maintenance targets are successfully achieved, thereby reducing the risk of surgical site infections and impaired wound healing.^104^, ^105^ A recent kidney function panel and trough levels for tacrolimus and/or sirolimus are required preoperatively to ensure therapeutic dosing. Addressing post-transplant anemia is a priority. Optimizing hemoglobin levels preoperatively can prevent the need for blood transfusions and subsequent human leukocyte antigen sensitization. If transfusion is unavoidable, leukocyte-reduced blood products must be used to minimize the risk of developing donor-specific antibodies.

Although most immunosuppressants can delay wound healing, sirolimus carries a significantly higher risk.^106^ Comparing a sirolimus-mycophenolate mofetil-prednisone regimen to a tacrolimus-mycophenolate mofetil-prednisone regimen found a much higher incidence of wound healing complications in the sirolimus group (47% vs. 8%, *P* < 0.0001).^107^ If a patient is on sirolimus, the transplant nephrologist should be consulted before surgery to discuss potential temporary regimen adjustments.

### Intraoperative and Immediate Perioperative Considerations

Both general and neuraxial anesthesia have successfully been used in transplant recipients.^108^ Because of surgical denervation, the transplanted kidney exhibits diminished vasomotor autoregulation, making it highly sensitive to fluctuations in systemic blood pressure.^109^ Avoiding severe intraoperative hypotension is therefore critical to prevent graft ischemia. Antibiotic prophylaxis should be administered per standard surgical infection prevention protocols; broad coverage for opportunistic infections is not required unless clinically indicated.^110^

It remains unclear if stress-dose steroids are warranted perioperatively for patients taking steroids as part of their immunosuppressive regimen. Although the overall incidence of hypothalamic-pituitary-adrenal axis suppression is as low as 1%, many providers still increase the steroid dose perioperatively, especially for patients taking >20 mg prednisone per day. However, multiple reports show patients successfully undergoing surgery by simply continuing their chronic prednisone maintenance dose.^111^ In a prospective study of 40 kidney transplant recipients under significant physiological stress (e.g., surgery or sepsis), continuing maintenance prednisone (5–10 mg/d) resulted in no cases of adrenal insufficiency.^112^

### Postoperative Management

Close monitoring of immunosuppressant levels is mandatory in the perioperative period. When patients are nil-per-oral and cannot tolerate oral capsules, enteral, i.v., or sublingual formulations are appropriate alternatives. If the enteral route is viable, a suspension can be administered via nasogastric tube. For sublingual administration, a dose reduction targeting approximately 50 percent of the total daily oral dose may serve as an initial starting point, but this conversion should be discussed with transplant team alongside rigorous trough-level monitoring and careful titration. Trough levels for tacrolimus and sirolimus should be measured 12 hours post-dose, immediately before the next scheduled administration.^105^, ^113^, ^114^ Drug-drug interactions are common in this population because of hepatic metabolism via the cytochrome P450 3A4 pathway. Potent inhibitors, such as azole antifungals and calcium channel blockers like nifedipine, increase calcineurin inhibitor levels, whereas enzyme inducers such as rifampin and phenytoin decrease them. Macrolide antibiotics also impact tacrolimus concentrations—erythromycin and clarithromycin elevate levels, whereas azithromycin does not demonstrate this interaction.^115^, ^116^

## Future Directions

Ongoing trials are evaluating optimal preoperative dialysis scheduling for kidney failure (NCT07339202). Future efforts must prioritize risk prediction models tailored for CKD, as general surgical scores lack necessary granularity. Simultaneously, integrating machine learning algorithms and novel biomarker panels, such as measuring insulin-like growth factor-binding protein 7 and tissue inhibitor of metalloproteinases-2 to trigger KDIGO care bundles involving fluid optimization, perfusion maintenance, and nephrotoxin discontinuation in electronic medical records can mitigate AKI severity.^117^, ^118^ Finally, formalizing perioperative nephrology consultation services is essential to integrate nephrologists into multidisciplinary care pathways.^119^ Future investigative priorities and key knowledge gaps are summarized in Supplementary Table S2.

## Conclusion

With the increasing prevalence of CKD, more patients will require surgery. Providers must look beyond standard cardiac risk to evaluate perioperative kidney risk using validated models and individualized clinical strategies. Figure 1 provides a comprehensive summary of these perioperative recommendations. Optimizing these complex scenarios requires close multidisciplinary coordination among internists, surgeons, cardiologists, anesthesiologists, nephrologists, and transplant specialists, ensuring all stakeholders implement evidence-based approaches to protect kidney outcomes.

**Figure 1:**
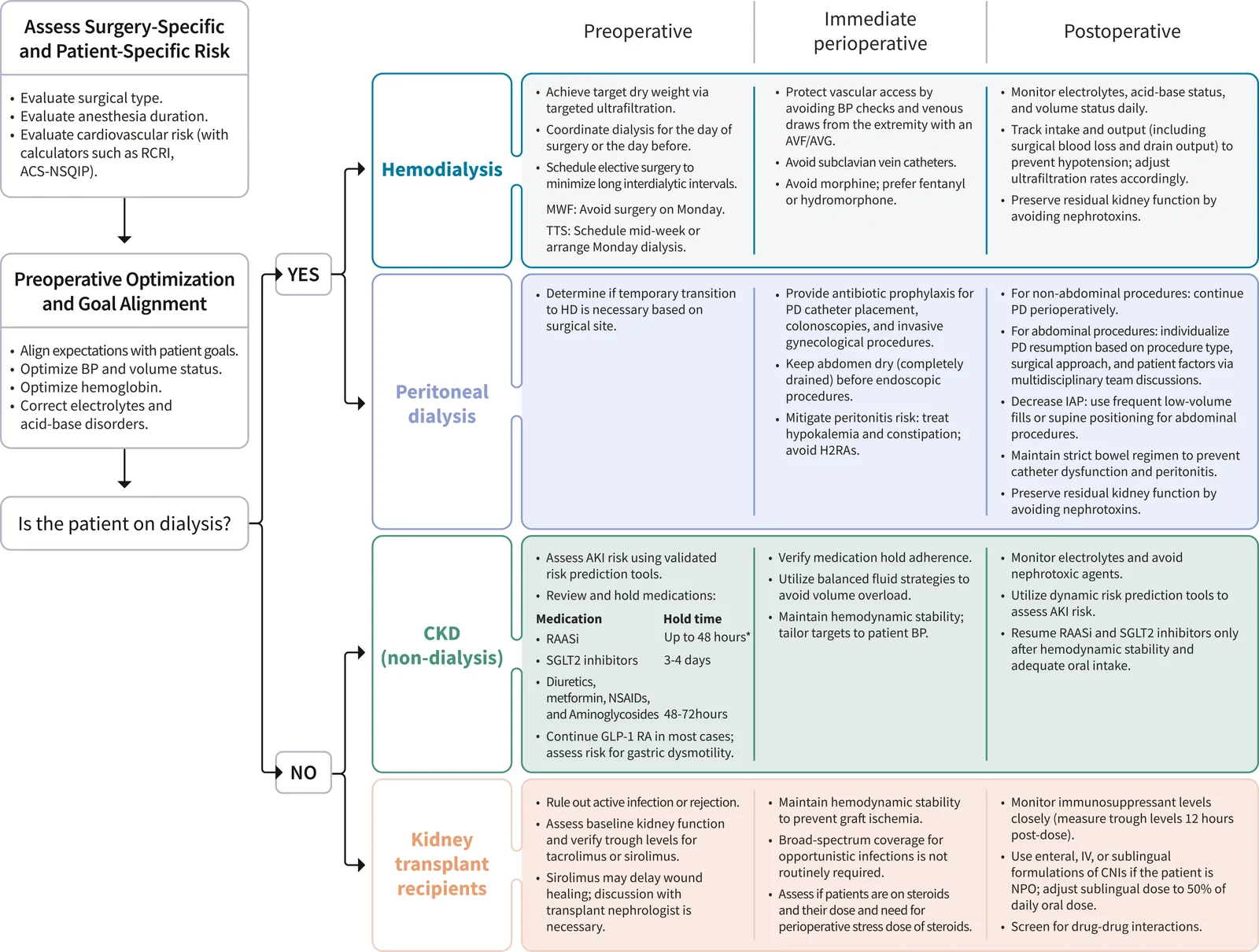
Proposed risk mitigation approach to perioperative management of patients with kidney disease. This diagram represents the authors’ synthesis of data discussed in the narrative and does not reflect an evidence-based guideline. ∗RAASi hold duration (up to 48 hours) should be individualized based on clinical indication, blood pressure control, and surgical risk profile. ACS-NSQIP, American College of Surgeons National Surgical Quality Improvement Program; AKI, acute kidney injury; AVF/AVG, arteriovenous fistula/arteriovenous graft; BP, blood pressure; CKD, chronic kidney disease; CNIs, calcineurin inhibitors; GLP-1 RA, glucagon-like peptide-1 receptor agonist; H2RAs, H2-receptor antagonists; HD, hemodialysis; IAP, intra-abdominal pressure; i.v., intravenous; MWF/TTS, Monday-Wednesday-Friday/Tuesday-Thursday-Saturday; NPO, nil per os (nothing by mouth); NSAIDs, nonsteroidal anti-inflammatory drugs; PD, peritoneal dialysis; RAASi, renin-angiotensin-aldosterone system inhibitors; RCRI, Revised Cardiac Risk Index; SGLT2 sodium-glucose cotransporter-2 inhibitors.

## Disclosure

All the authors declared no competing interests.

## Supplementary Material

Supplementary file (PDF) **Table S1.** Studies on perioperative management of hypertension. **Table S2.** Key knowledge gaps and future research priorities in perioperative nephrology.

## Footnotes

Supplementary file (PDF)
**Table S1.** Studies on perioperative management of hypertension. **Table S2.** Key knowledge gaps and future research priorities in perioperative nephrology.
