# Supplementary material for: Perioperative Risk Assessment and Management in Patients With CKD, Dialysis Dependence, and Kidney Transplantation
Source: Kidney Int Rep. 2026 Oct 10;12(1):107137. doi: 10.1016/j.ekir.2026.107137 (PMC13761351; doi:10.1016/j.ekir.2026.107137)
Supplement: Supplementary file 1 [file mmc1.pdf]

Study	Design	Participants	Results
POISE-3 trial	Randomized controlled trial	7,490 patients at risk for vascular complications undergoing noncardiac surgery (including 1,484 patients with pre-existing CKD). Patients were randomized to a hypotension-avoidance strategy (targeting an intraoperative MAP of 80 mmHg) or a hypertension-avoidance strategy (targeting a MAP of 60 mmHg).	-Both strategies resulted in a similar incidence of major vascular complications, and there was no significant difference in the incidence of postoperative AKI<br>-Hypotension-avoidance strategy, which included holding RAASi, resulted in a lower incidence of intraoperative hypotension compared to the hypertension-avoidance strategy, yet it did not significantly reduce the risk of postoperative AKI showing identical rates in high-risk CKD subgroups (17.4% vs. 16.8%).
Continuation vs Discontinuation of RASi Before Major Noncardiac Surgery (Stop-or-Not) trial	Randomized controlled trial	2,222 patients (including 198 patients with a history of CKD) were randomized to continue RAASi until the day of surgery or to discontinue RASIs 48 hours prior to surgery.	Episodes of hypotension during surgery occurred in 54% of the RAASi continuation group compared to 41% in the RAASi discontinuation group. However, both groups had similar rates of AKI (11%), with findings consistent across CKD subgroups (risk ratio 1.26 [95% CI, 0.87–1.82]; P=0.23 interaction).

Supplementary Table S1: Studies on perioperative management of hypertension
Key: POISE: PeriOperative ISchemic Evaluation;; RAAS, renin-angiotensin-aldosterone system; MAP: Mean arterial pressure; AKI: Acute kidney injury.

Domain / Category	Priority Research Question / Knowledge Gap
Incidence of AKI	While established risk thresholds exist for high-risk procedures like cardiac and vascular surgery, procedure-specific AKI incidence remains unestablished across the broader spectrum of general and specialized surgeries. Future prospective investigations using uniform KDIGO criteria are needed to define precise AKI incidence across diverse surgical procedures in patients at different stages of CKD and in kidney transplant recipients.
Risk Stratification Tools	Prospective validation and recalibration of perioperative risk stratification tools are required specifically for advanced CKD and kidney transplant populations.
Procedural and Dialysis Timing & Anticoagulation	Optimal time intervals and management strategies for intra-dialytic heparinization relative to elective or emergency surgery remain undefined, requiring dedicated prospective evaluation to balance circuit clotting risks against surgical bleeding complications.
Modality Comparisons	Comparative analyses are needed to evaluate whether advanced extracorporeal modalities, such as hemodiafiltration versus standard hemodialysis, offer distinct perioperative morbidity or mortality benefits.
RAASi Perioperative Strategies	High-quality prospective trials are required to evaluate perioperative RAASi continuation versus cessation strategies across distinct clinical indications, including uncomplicated hypertension, HFrEF, proteinuric CKD, and resistant hypertension.
SGLT2i Emergency Protocols	Large-scale prospective evaluations are needed for SGLT2i continuation versus cessation strategies in emergency surgical settings, focusing specifically on euglycemic DKA risk profiles in advanced CKD across distinct clinical indications such as heart failure, diabetic kidney disease, and proteinuric CKD.

Supplementary Table S2. Key knowledge gaps and future research priorities in perioperative nephrology.

Key: AKI, acute kidney injury; CKD, chronic kidney disease; DKA, diabetic ketoacidosis; HFrEF, heart failure with reduced ejection fraction; KDIGO, Kidney Disease: Improving Global Outcomes; RAASi, renin-angiotensin system inhibitor; SGLT2i, sodium-glucose cotransporter-2 inhibitor.
